# Spontaneous chylous cardiac tamponade: a case report

**DOI:** 10.1186/1749-8090-5-11

**Published:** 2010-03-17

**Authors:** Nikolaos Barbetakis, Christos Asteriou, Dimitrios Konstantinou, Dimitrios Giannoglou, Christodoulos Tsilikas, Georgios Giannoglou

**Affiliations:** 1 Cardiothoracic Surgery Department, Theagenio Cancer Hospital, Al. Symeonidi 2, Thessaloniki, Greece, 54007; 2 Cardiology Department, Aristotle University, AHEPA Hospital, S. Kiriakidi 1, Thessaloniki, Greece, 54630

## Abstract

**Background:**

Chylous cardiac tamponade is a rare condition with little known cause.

**Case presentation:**

A case of an otherwise healthy woman who admitted with dyspnea and palpitations is presented. She had a history of a painful flexion-hyperextension of the spine. Diagnostic evaluation proved a chylous pericardial effusion with a disruption of the anterior longitudinal spinal ligament. Video-assisted thoracic surgery with mass supradiaphragmatic ligation of the thoracic duct and pericardial window formation was carried out successfully and resulted in the complete cure of the patient's condition.

**Conclusion:**

Chylous pericardial effusion and subsequent tamponade is a rare entity. Endoscopic surgery is offering a safe and effective treatment.

## Background

Chylous pericardial effusion may occur following cardiothoracic surgery or in association with congenital lymphangiomatosis. Other causes may include chest trauma, mediastinal irradiation, malignant diseases, filariasis and thrombosis of the subclavian vein and superior vena cava. Primary chylopericardium has also been described, most commonly in children and young adults [1]. Thirty three cases were identified from 31 articles through a systematic literature search.

Herein, a case of spontaneous chylous cardiac tamponade which was successfully treated by video-assisted thoracic surgery (VATS) is reported.

## Case presentation

**Figure 1:**
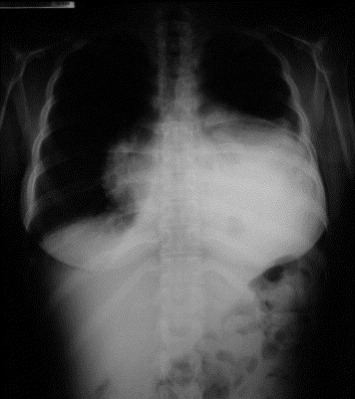
**Preoperative chest x-ray demonstrating cardiac enlargement due to pericardial effusion**.

A 41-year-old female was admitted to our hospital with shortness of breath for about 24 hours. There was no significant past medical or surgical history except for the fact that she experienced a painful hyperextension of the spine the previous morning, during routine physical exercise. A subsequent chest x-ray showed enlargement of the cardiac silhouette (Figure 1).

**Figure 2:**
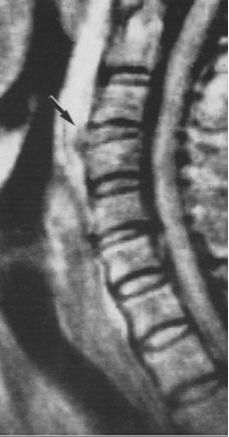
**Magnetic resonance imaging was consistent with a disruption of the anterior longitudinal ligament and anterior protrusion of the intervertebral disc (black arrow)**.

A transthoracic echocardiogram demonstrated a large pericardial effusion, with right ventricular collapse consistent with cardiac tamponade physiology. An urgent therapeutic pericardiocentesis was performed and 1200 ml of milky fluid was removed and an 8 Fr drain was left in place. The laboratory results of the fluid revealed the following: triglycerides 550 mg/dl, cholesterol 110 mg/dl, total proteins 4.6 g/dl, glucose 85 mg/dl. The diagnosis of chylopericardium was established. Cytology stains and cultures were all unremarkable. Blood tests for rheumatologic, endocrinologic and autoimmune disorders were normal. Tests for bacterial, fungal, mycobacterial and viral infections were also conducted and found negative. Chest, abdomen and brain scans were normal. No evidence of lymphadenopathy was noted. Despite the absence of severe symptomatology concerning the spine injury a magnetic resonance imaging of the thoracic spine was ordered and was consistent with a disruption of the anterior longitudinal ligament and anterior protrusion of the intervertebral disc (Figure 2).

A daily output of 350 ml of pericardial fluid led us to start total parenteral nutrition, subcutaneous octreotide and no oral feedings for 7 days. These conservative measures proved to be unsuccessful because the rate of the pericardial drainage did not decrease. The patient also underwent, a bipedal lymphangiography which showed no anatomic abnormalities of the thoracic duct and no leakage. Under these circumstances the patient was addressed for thoracic surgical evaluation. The patient finally underwent a video-assisted supradiaphragmatic mass ligation of the thoracic duct and creation of a pleuropericardial window through a low right mini thoracotomy. Ligation of the thoracic duct together with all the adjacent soft tissue between the esophagus, the azygos vein and the aorta was performed. The patient was recovered uneventfully for both spine injury and tamponade. There has been no recurrence of the pericardial effusion for 12 months.

## Discussion

Chylopericardium is sometimes a consequence of thoracic and cardiac surgery. It may also occur as a result of chest trauma, mediastinal neoplasms, mediastinal tuberculosis, mediastinal radiotherapy, and thrombosis of the subclavian vein [2].

Idiopathic chylopericardium is a rare entity. It was first reported in 1886 by Hasebrock. The term primary isolated chylopericardium was first reported by Groves and Effler in 1954 [3].

Its precise etiology still remains unknown. Primary chylous pericardial effusions result from retrograde flow through abnormal lymphatics into rich pericardial plexus. Such abnormal lymphatic channels may represent lymphangiomas or they may be a part of larger lymphatic tumors [4]. Several mechanisms have been proposed to explain the development of chylous pericardial effusions. Most secondary effusions are caused by interruption of the thoracic duct by surgery, inflammation or non lymphatic tumor. Normal lymphatic valves prevent chylous reflux into the pericardial plexus even after ligation of the thoracic duct proximal to the pericardial tributaries, unless concurrent superior vena caval ligation prevents collateral flow. Blunt chest trauma may rupture lymphatic valves by precipitously elevating intrathoracic pressure [5]. This mechanism caused by the flexion - hyperextension movement of the thoracic spine, could be the underlying mechanism in this case. The problem is that pedal lymphoscintigraphy did not prove any communication between thoracic duct or branches and pericardial sac.

Symptoms depend on the importance of the effusion and on compression of the cardiac cavities. Chronic effusions may remain asymptomatic for a long time. Whenever cardiac compression occurs symptoms are those observed with tamponade and include: exertional dyspnea, chest pain, fatigue and palpitations. Asymptomatic pericardial effusions are usually diagnosed on routine chest x-ray, echocardiography, computerized tomography scan or magnetic resonance imaging.

Chylopericardium is usually diagnosed by pericardiocentesis that shows the presence of chylous fluid with high triglyceride level. Pathological analysis demonstrates white-yellow chylous fluid with numerous foamy cells and fat globules shown by Sudan III staining [6]. Also noted are extra-cellular fat droplets and predominance of lymphocytes [7].

Many diagnostic modalities have been described, including observation of Sudan III dye distribution into the pericardial cavity after oral intake of Sudan III dye, lymphangioscintigraphy, lymphangiography and evaluation of chest radioactivity after an oral dose of 131I-triolein. All of these methods are used to ascertain the cause of the chylous pericardial effusion [8]. According to the literature, demonstrable abnormalities of thoracic lymphatic vessels were present in 4 out of 5 patients who presented with cardiac tamponade and in 1 of 2 patients who developed tamponade after pericardiocentesis [4].

Non surgical management includes dietary regimen with nothing per os or medium chain triglycerides, total parenteral nutrition and subcutaneous octreotide. However this conservative treatment alone is associated with reaccumulation of fluid [9].

Surgical treatment has been proposed to halt recurrence and progression for cardiac tamponade. Surgical modalities include pericardial window formation, thoracic duct ligation and pericardial-peritoneal shunting. The success of combined thoracic duct ligation above the diaphragm and pericardial window has been documented [9]. Furrer and colleagues described the first successful thoracoscopic approach to primary chylopericardium [1]. The authors mentioned a mass ligation of all tissues situated between the azygos vein, vertebral body and descending aorta. This kind of approach was used in our case with excellent results. The left-sided approach has some disadvantages because in the lower thoracic cavity, the thoracic duct is located to the right of the descending aorta. This prevents easy access to the duct when entering from the left hemithorax. The VATS procedure is being used increasingly and is associated with less postoperative pain and pulmonary dysfunction [10].

## Conclusions

In conclusion, a rare case of chylous cardiac tamponade probably related to a previous thoracic spine flexion-hyperextension injury was presented. Lymphoscintigraphy failed to prove communication between thoracic duct and pericardial sac. Video-assisted thoracic surgery with pericardial window formation and supradiaphragmatic mass ligation of the thoracic duct was curative.

## Competing interests

The authors declare that they have no competing interests.

## Authors' contributions

NB, CA, DK, DG and CT took part in the care of the patient and contributed equally in carrying out the medical literature search and preparation of the manuscript. GG participated in the care of the patient and had the supervision of this report. All authors approved the final manuscript.

## Consent

Written informed consent was obtained from the patient for publication of this case report and accompanying images. A copy of the written consent is available for review by the Editor-in-Chief of this journal.

## References

[B1] Furrer M Hopf M Ris HB Isolated primary chylopericardium: treatment by thoracoscopic thoracic duct ligation and pericardial fenestration J Thorac Cardiovasc Surg 1996 112 1120 1121 10.1016/S0022-5223(96)70119-8 8873745

[B2] Mehrotra S Peeran NA Bandyopadhyay A Idiopathic chylopericardium. An unusual cause of cardiac tamponade Tex Heart Inst J 2006 33 249 252 16878639 PMC1524687

[B3] Groves LK Effler DB Primary chylopericardium N Engl J Med 1954 250 520 523 13144986 10.1056/NEJM195403252501206

[B4] Dunn RP Primary chylopericardium: a review of the literature and an illustrated case Am Heart J 1975 89 369 377 10.1016/0002-8703(75)90088-5 1114965

[B5] Gallant TE Hunziker RJ Gibson TC Primary chylopericardium: The role of lymphangiography Am J Roentgenol 1977 129 1043 1045 10.2214/ajr.129.6.1043 413356

[B6] Wang CH Yen TC Ng KK Lee CM Hung MJ Cherng WJ Pedal (99m) Tc-sulfur colloid lymphoscintigraphy in primary isolated pericardium Chest 2000 117 598 601 10.1378/chest.117.2.598 10669713

[B7] Akamatsu H Amano J Sakamato T Suzuki A Primary chylopericardium Ann Thorac Surg 1994 58 262 266 8037546 10.1016/0003-4975(94)91124-x

[B8] Dib C Tajik AJ Park S Kheir ME Khanderia B Mookadam F Chylopericardium in adults: a literature review over the past decade (1996-2006) J Thorac Cardiovasc Surg 2008 136 650 656 10.1016/j.jtcvs.2008.03.033 18805268

[B9] Sakata S Yoshida I Otani Y Ishikawa S Morishita Y Thoracoscopic treatment of primary chylopericardium Ann Thorac Surg 2000 69 1581 1582 10.1016/S0003-4975(00)01185-1 10881850

[B10] Kirby TJ Mack MJ Landreneau RJ Rice TW Lobectomy--video assisted thoracic surgery versus muscle-sparing thoracotomy. A randomized trial J Thorac Cardiovasc Surg 1995 109 997 1001 10.1016/S0022-5223(95)70326-8 7739262

